# Design, Analysis and Experiment of a Bridge-Type Piezoelectric Actuator for Infrared Image Stabilization

**DOI:** 10.3390/mi12101197

**Published:** 2021-09-30

**Authors:** Mengxin Sun, Yong Feng, Yin Wang, Weiqing Huang, Songfei Su

**Affiliations:** 1 Department of Mechanical Engineering, Nanjing Institute of Technology, Nanjing 211167, China; fengyong007@sina.com (Y.F.); lele492112017@56.com (S.S.); 2 College of Mechanical Engineering and Automation, Huaqiao University, Quanzhou 361021, China; yin.wangyin@hqu.edu.cn; 3 School of Mechanical and Electric Engineering, Guangzhou University, Guangzhou 510006, China; mehwq@nuaa.edu.cn

**Keywords:** piezoelectric actuator, infrared image stabilization, real-time control

## Abstract

Piezoelectric actuators are widely used in the optical field due to their high precision, compact structure, flexible design, and fast response. This paper presents a novel piezoelectric actuator with a bridge-type mechanism, which can be used to stabilize the images of an infrared imaging system. The bridge amplification mechanism is used to amplify the actuation displacement, and its structural parameters are optimized by the response surface method. The control strategy of the image stabilization system is formulated, and the overall structure of the infrared image stabilization system is designed according to the principle of image stabilization and the control strategy. The prototype was fabricated and verified by a series of experiments. In the test, the laminated piezoelectric ceramics are used as the driving element, and its maximum output displacement was about 17 μm under a voltage of 100 V. Firstly, the performance of the piezoelectric amplification mechanism was tested, and the maximum displacement of the piezoelectric micro-motion mechanism was 115 μm. The displacement amplification ratio of the mechanism was 5.7. Then, the step distance and response time of the micro-displacement mechanism were measured by inputting the stepping signal. When the input voltage increased to 3 V, 5 V, and 7 V, the stepping displacements of the mechanism were 2.4 μm, 4.1 μm, and 5.8 μm. Finally, the image stabilization effect of the designed mechanism was verified by imaging timing control and feedback signal processing.

## 1. Introduction

An infrared imaging system usually faces a complex and difficult working environment. For example, military UAVs (unmanned aerial vehicles) or precision strike weapons may be disturbed by air turbulence in flight, causing infrared detectors to experience large torque or high-frequency vibration in the imaging process, thus resulting in blurred, distorted, or ghosting images from which the target information cannot be effectively obtained [1,2,3,4]. Therefore, it is urgent to introduce an image stabilization mechanism in the imaging system to compensate for the imaging error and improve the imaging accuracy.

Image stabilization technology is widely used in reconnaissance, aerial survey, and photographic systems [5,6,7,8,9]. The existing image stabilization technology can be categorized into three types based on its principle: mechanical image stabilization [10], electronic image stabilization [11,12,13], and optical image stabilization [14,15,16]. Mechanical image stabilization relies on a gyroscope to sense the vibration of the target. Meanwhile, the servo system is used for feedback and to compensate for the relative motion of the imaging system. As revealed by the previous research, the mechanical image stabilization system with a platform usually features a complex structure and large size, for which it can only get high accuracy and real-time performance under low frequencies. Electronic image stabilization is a method to realize image stabilization by using image processing technology to process the blurred image and compensate for the inter frame jitter of the image. In recent years, many researchers have made efforts to improve the quality, speed, and accuracy of electronic image stabilization [17,18,19,20]. Chang et al. proposed a novel motion estimation algorithm for image stabilization, which was effective at reducing motion-matching manipulation [21]. Marcos et al. suggested a technique for integrating local features of video stabilization. As demonstrated by the experimental results, the combination method was effective in achieving digital stabilization [22]. Generally speaking, electronic image stabilization technology is only reliant on algorithm and image processing. It shows various advantages such as flexible mode, small size, and low cost. Nevertheless, its inherent characteristics restrict its ability to adapt to high frequency and large amplitude vibration. Optical image stabilization refers to the addition of one or more movable optical elements in the optical system of image stabilization instrument. When the output image is blurred by jitter, the optical elements are fine-tuned to compensate for imaging errors, thus achieving image stabilization [23,24,25]. The existing optical image stabilization technology is characterized by high accuracy and a large adjustment range, for which it has played a significant role in various low-speed and low-frequency applications. Ingo Walter et al. presented a method of image motion compensation using micro-mechanical devices for an imaging system [26]. Akira Heya et al. proposed a new 3-DOF electromagnetic actuator, which is capable of being controlled by a simple system to ensure the image stabilization [27]. Nevertheless, the optical image stabilization technology for infrared imaging systems remains a challenge in complex environments.

Allowing for the common problems with the existing image stabilization methods, a piezoelectric actuator has been identified as an ideal intelligent structure in the optical element with various advantages including high accuracy, compact size, fast response, power-off self locking, and non-electromagnetic interference. In recent years, piezoelectric actuators are widely used in microscopes, optical scanners, and other optical devices [28,29,30,31,32]. For instance, Li proposed a novel type of piezoelectric actuator with features of compact size, high precision, light weight, and self locking to drive an aperture, which achieves the integration of the aperture and actuator [33]. Furthermore, Michael reported an actuation structure with eight unimorph piezoelectric actuators for out-of-plane micro-lens movement. The reported mechanism demonstrates higher resonant frequency for which it is suited for faster actuation [34].

For these reasons, a new infrared image stabilization based on a novel piezoelectric actuator is proposed in this paper. The paper is structured as follows. Initially, the design concepts and principles of the proposed image stabilization system are presented and elaborated. Subsequently, an equivalent stiffness model of the actuator and control strategy of the system is constructed for the purpose of parameter optimization and kinetic analysis. In addition, a series of experiments are performed to characterize the performance and to validate the feasibility of the principle of the image stabilization mechanism. Finally, a conclusion is reached. Compared with other reported piezoelectric actuators, the piezoelectric actuator with bridge amplification in this paper can not only complete the high-precision (Submicron scale) actuation but also realize the adjustment of a large stroke (more than 100 μm). It also has fast response (millisecond level), which can better meet the real-time image stabilization requirements of the infrared imaging system. Through parameter optimization, appropriate stiffness, and output displacement of the image stabilization mechanism can be provided for different image stabilization scenes. The possible limitation of the mechanism is that after the infrared imaging system generates heat in use, it may lead to changes in the properties of piezoelectric ceramics, which will affect the adjustment accuracy of the system.

## 2. Design of the Actuator

### 2.1. Design Concepts and Principles

The working principle of image stabilization is as follows. A gyroscope is employed to collect real-time signals released by the jitter of the camera during the shooting process. Based on the calibration relationship between the angular velocity and offset pixels in the image stabilization period, the piezoelectric mechanism is applied to drive the lens to compensate for imaging offset. Among them, the control module needs to convert the feedback signal of the gyroscope at a certain time into the driving signal input to the piezoelectric ceramics.

**Figure 1:**
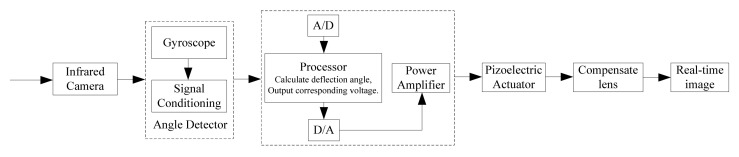
The control flow chart of the optical image stabilization.

The objective of the infrared image stabilization system designed in this paper is to stabilize the image by controlling the motion of the compensating lens driven by the piezoelectric actuator. Figure 1 presents the control flow chart of the optical image stabilization in the infrared device. The control process is open-loop. The image stabilization control system is based on the control program written by the PSoC development system. With the assistance of the A/D and D/A conversion module of the chip, it receives the voltage signal of the gyroscope output angular velocity and converts it into digital quantity, calculates the deflection angle at the moment t, and then applies the voltage on laminated piezoelectric ceramics based on the relationship between deflection angle and the driving voltage. The analog voltage signal sent by the controller is calculated and connected to the piezoelectric stacks through the power amplifier, thus initiating the compensation mechanism. Finally, the real-time image is obtained.

In this principle, the piezoelectric mechanism drives the compensating lens to achieve real-time high-precision micro-displacement action. 

### 2.2. Design of the Piezoelectric Actuator

The image stabilization system puts forward not only the requirement of high precision and fast response but also the requirements of small size and large stroke. The proposed piezoelectric mechanism is mainly used to adjust the compensation lens in the infrared imaging system to realize the image stabilization function. In imaging, we should not only ensure high accuracy but also provide enough stroke. In the infrared imaging system adopted in this paper, adjusting one pixel corresponds to moving the compensate lens a distance of 10 μm. In order to keep accurate position and less cumulative error in adjustment, it is necessary to improve the stepping accuracy to the submicron level at least. On the other hand, when the external disturbance is large, the imaging offset may be more than a few pixels, so it is necessary that the lens can move stably by about 100 μm. At the same time, it is also necessary to ensure millimeter-level response speed.

If the piezoelectric motor is used to drive the guide rail by friction, it is difficult to ensure the stable output performance under load. If laminated piezoelectric ceramics are used for direct output, the stroke is difficult to meet the requirements when preload is applied. Therefore, it is necessary to adopt a flexible amplification micro-displacement mechanism with certain stiffness, which can not only ensure the stable output performance but also meet the requirements of high precision and large stroke.

**Figure 2:**
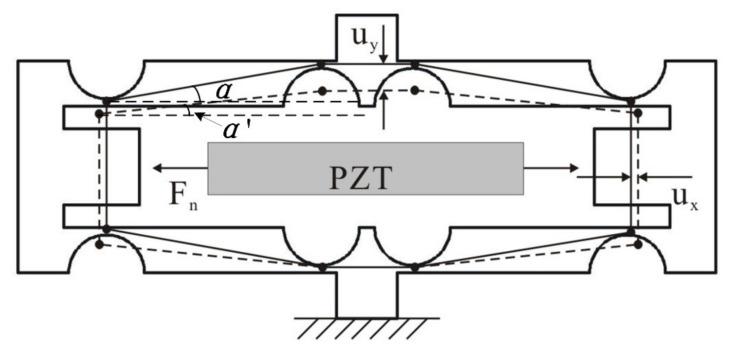
Piezoelectric micro-displacement actuator.

According to the demand, a piezoelectric micro-displacement actuator is designed with flexible structure based on the bridge amplification principle, as shown in Figure 2. The laminated piezoelectric ceramics (PZT) output driving force *F*_n_ after applying voltage, and the amplifying mechanism deforms under the action of the driving force. According to the geometric relationship, the theoretical magnification ratio of the bridge amplification mechanism can be calculated as follows.

(1) $$\beta =\frac{u_{y}}{u_{x}}=\frac{\sin\alpha -\sin\alpha^{\prime }}{\cos\alpha^{\prime }-\cos\alpha }\approx \frac{1}{\tan\alpha }$$

where *u*_x_ is input displacement, and *u*_y_ is output displacement.

**Figure 3:**
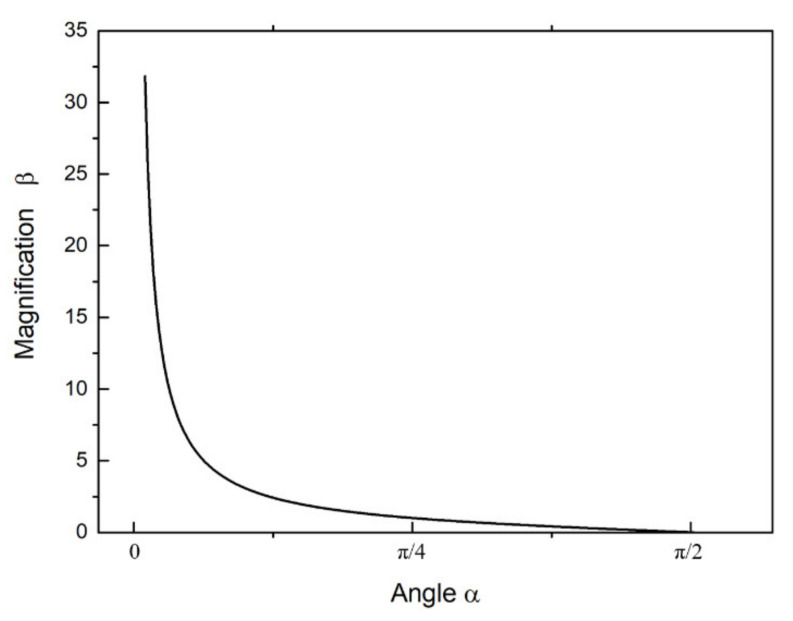
The relationship between the magnification and angle.

According to Formula (1), it can be seen that the magnification ratio of the bridge amplification mechanism is only related to the angle, and the relationship curve is shown in Figure 3. It can be seen that when the angle is close to 0, the magnification ratio reaches infinity theoretically, and the small change of the angle will cause a great change in the magnification ratio. When the angle is close to π/2, the magnification ratio is almost zero, and the change rate of the magnification ratio is close to zero. Therefore, when designing the angle, it needs to consider the influence of the angle on the magnification.

**Figure 4:**
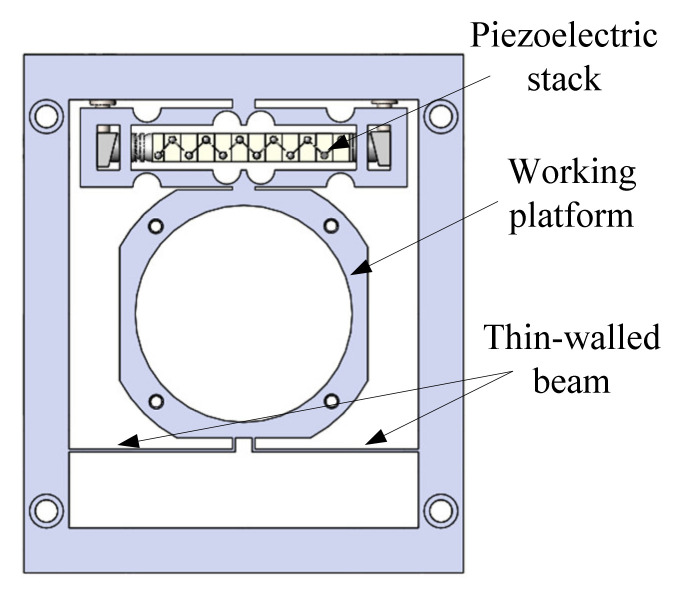
The structure of the stabilization mechanism.

On the basis of the bridge-type micro-displacement amplification mechanism, the design scheme of an image stabilization mechanism is proposed. The structure of the stabilization mechanism is shown in Figure 4. The mechanism consists of a piezoelectric actuator, two thin-walled beams, and a working platform. The working platform is set for installing the compensation lens. The thin-walled beam acts as a guiding mechanism to avoid displacement deviation in the process of motion.

**Figure 5:**
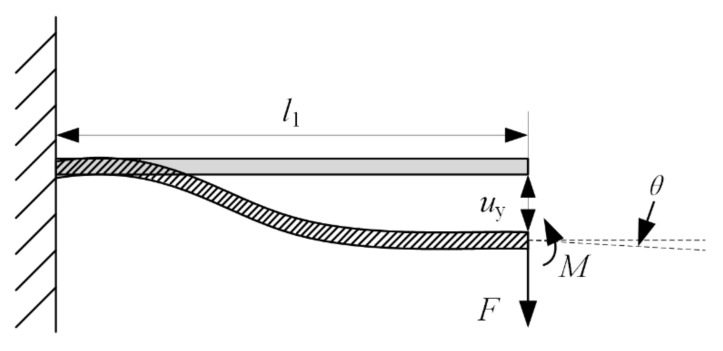
The model of the thin-walled beam.

The thin-walled beam used in the structure can be regarded as a cantilever beam, and one end of the beam is fixed while the other end is loaded force *F* and counterclockwise moment *M*. The stress and deformation of the thin-walled beam are shown in Figure 5. The deflection and rotation angle under force and moment can be obtained by the superposition method, respectively.

Under the force *F*, the deflection and rotation angle of the cantilever beam are calculated as follows.

(2) $$\left\{\begin{array}{c} u_{y}^{F}=\frac{Fl_{1}{}^{3}}{3EI}\\ \theta^{F}=\frac{Fl_{1}{}^{2}}{2EI} \end{array}\right.$$

Under the moment *M*, the deflection and rotation angle of the cantilever beam are calculated as follows.

(3) $$\left\{\begin{array}{c} u_{y}^{M}=-\frac{Ml_{1}{}^{2}}{2EI}\\ \theta^{M}=-\frac{Ml_{1}}{EI} \end{array}\right.$$

where *l*_1_ is the length of thin-walled beams, *E* is modulus of elasticity of the cantilever beam, *I* is the inertia moment of the cantilever beam.

Since the rotation angle at the loading end of the thin-walled beam is zero, it can be calculated as follows.

(4) $$\theta^{F}+\theta^{M}=0$$

(5) $$M=Fl_{1}/2$$

According to Hooke’s law, the equivalent stiffness of a single straight beam can be calculated as follows:

(6) $$k_{e}=\frac{Ebt^{3}}{l_{1}{}^{3}}$$

where *b* is the breadth beam, and *t* is the thickness of beam.

**Figure 6:**
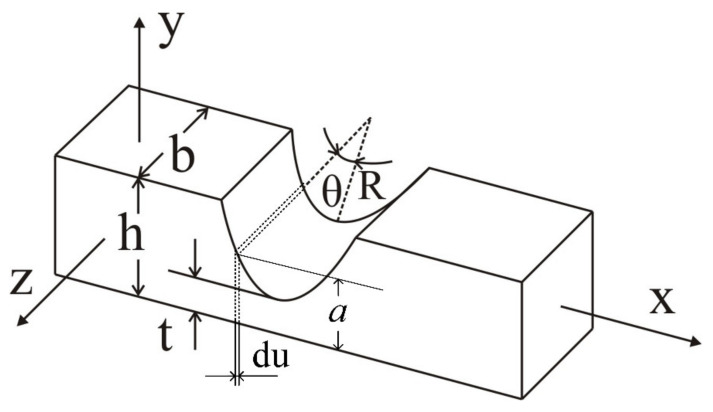
The structure of the circular flexible hinges.

The bridge-type amplification mechanism in this paper adopts circular flexible hinges, and its structure is shown in Figure 6. The Paros and Weisbord methods can be used to analyze the shape, force, and deformation of the flexure hinge. According to the size given in the figure, assuming that the left end of the flexure hinge is fixed and the right end is applied moment *M*_z_, the rotation angle at the right end of the flexure hinge is *α*.

To analyze conveniently, the micro-element is intercepted at the center angle *θ*, and the size of the micro unit is *a* × *b* × d*u*. The height *a* of the micro-element is

(7) a=t+R−Rcosθ.

Under the moment *M*_z_, the rotation angle of the micro-element along the axis *z* is as follows

(8) $$d\alpha_{z}=\frac{M_{z}}{EI_{z}}du$$

where *E* is module of elasticity, and *I*_z_ is the inertia moment along axis *z* of the flexure hinge.

Since each component of the displacement amplification mechanism produces elastic deformation at the flexure hinge only, the other parts can be considered as rigid. The stiffness of the flexure hinge around the axis is as follows [35].

(9) $$\begin{array}{l} k_{e}{}^{\prime }=\frac{M_{z}}{\alpha_{z}}=\frac{Eb}{12\int_{-\frac{\pi }{2}}^{\frac{\pi }{2}}\frac{R\cos\theta }{{\left(t+R-R\cos\theta \right)}^{3}}d\theta }\\ =\frac{EbR^{2}}{6\left[\frac{2s^{3}\left(6s^{2}+4s+1\right)}{\left(2s+1\right){\left(4s+1\right)}^{2}}+\frac{12s^{4}\left(2s+1\right)}{{\left(4s+1\right)}^{5/2}}\cdot \mathrm{arc}tg\sqrt{4s+1}\right]} \end{array}$$

where *s* = *R*/*t.*

**Figure 7:**
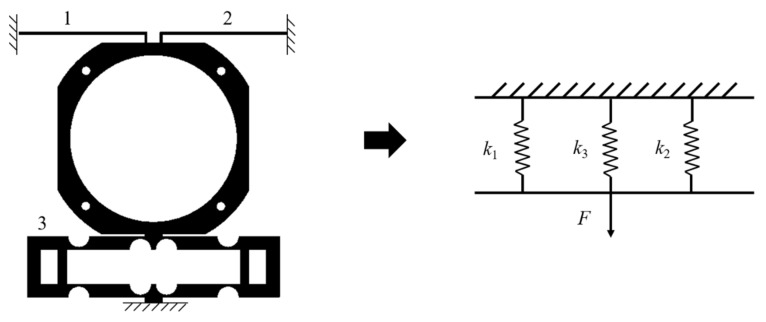
The equivalent stiffness model of the actuating mechanism.

The equivalent stiffness model of the single-layer actuating mechanism is shown in Figure 7. The bridge-type amplification mechanism is composed of eight flexible hinges. It can be considered that one side is connected by four hinges in series, and the left and right sides are connected in parallel. The equivalent stiffness of the single-layer mechanism is as follows.

### 2.3. Optimization Design

**Figure 8:**
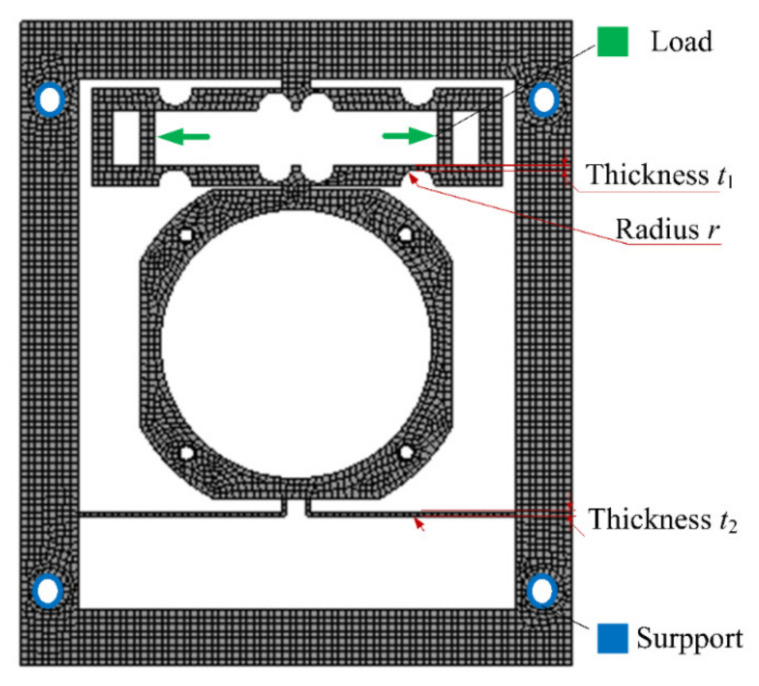
FEM model of the mechanism.

The finite element analysis software ANSYS was used for simulation analysis and parameter optimization of the mechanism, and the model meshing and constraint conditions are shown in Figure 8. The hex-dominant method is used in meshing when the body size is defined as 1 mm, and the element number is 151,234. In FEM models, the four mounting holes are set as fixed connections, and forces are loaded in two elongation directions of piezoelectric ceramic. Since the piezoelectric mechanism is operated in low frequency, static analysis is picked to obtain the results. According to the above theoretical analysis results, parameter optimization was carried out based on the response surface method with the thickness of the cantilever beam, the radius of the flexure hinge, and the thickness at the thinnest point of the flexure hinge as optimization variables, the displacement of frame as an objective function, and the maximum stress of the structure as constraint conditions.

**Figure 9:**
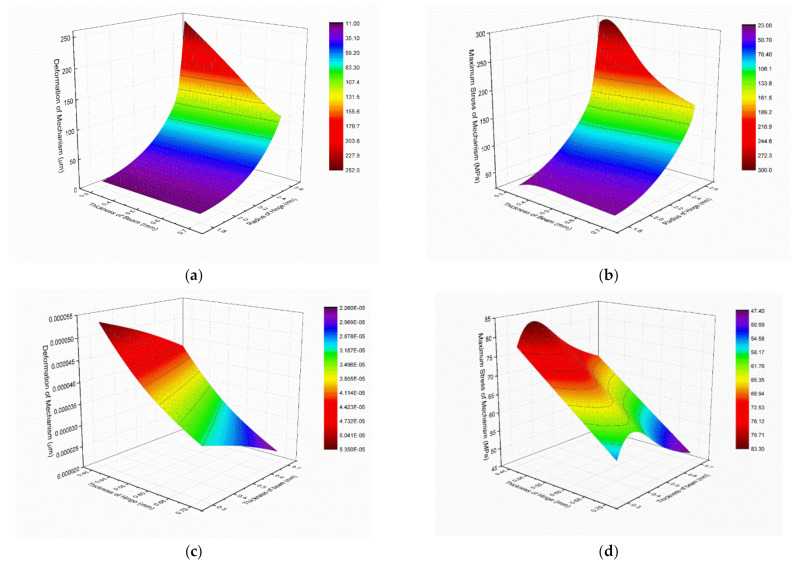
Relationship between structural parameters and output displacement/maximum stress. (**a**) Deformation versus thickness of beam and radius of hinge; (**b**) Maximum stress versus thickness of beam and radius of hinge; (**c**) Deformation versus thickness of hinge and beam; (**d**) Maximum stress versus thickness of hinge and beam.

According to the simulation results, the relationship between optimization parameters and system output displacement and structural stress can be obtained, as shown in Figure 9.

It can be seen that when the thickness of the beam, the thickness of the hinge, and the radius of the arc in the hinge increased, both the overall deformation and the maximum stress of the structure increased to different degrees. Under the limit of allowable stress of materials and machining requirements, the thickness of the beam, the thickness of the hinge, and the radius of the arc in the hinge were set to 0.5 mm, 2.5 mm, and 0.5 mm, respectively.

**Figure 10:**
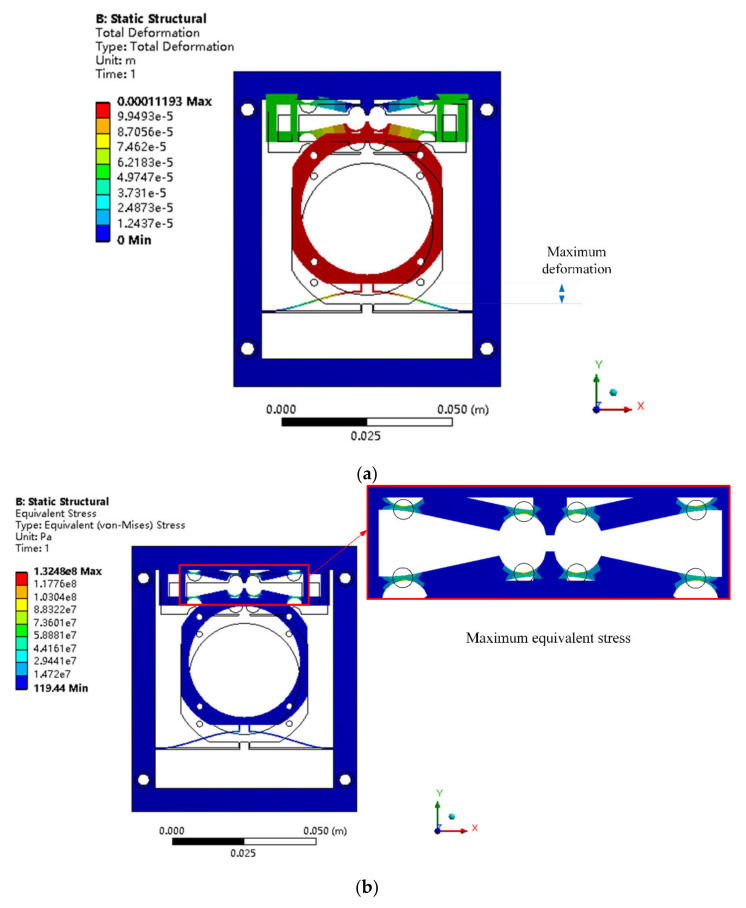
(**a**) Deformation and (**b**) equivalent stress of the FEM model of the piezoelectric mechanism.

Under the selected size parameters, the input/output validation of the structure was carried out, and the results are shown in Figure 10. When the piezoelectric actuator output displacement was 20 μm, the output displacement at the position of the loading lens in the mechanism reached 112 μm, the maximum stress was 132 MPa, and the amplification ratio was 5.6.

The main materials and dimensions of the piezoelectric mechanism are listed in Table 1.

### 2.4. Image Stabilization Strategy and Calculation

The key point of the image stabilization system strategy is the corresponding transformation relationship between the gyroscope feedback signal in the control module and the electrical signal input to the piezoelectric micro-module. The required diagonal field of view is 5°, the distance between the experimental object and the camera is 1 m, the camera resolution is 384 × 288, and the frame frequency is 50 Hz. The relation between the displacement of the compensation lens and the number of moving pixels is 10 μm/ pixel, and the travel of the compensation lens relative to the origin is ±60 μm.

The rotation angular velocity of the camera at time *t* under external interference is *ω*_x_, and the total field of view of lateral imaging is *α*.

The relation between the displacement of the compensation lens and the number of pixel deviations can be controlled as *e*. The view angle corresponding to each pixel in the jitter direction of the photosensitive element is defined as *k*_x_, and the pixel of migration image after interference *α*_x_ is

(10) $$a_{x}=\int_{0}^{t}\omega_{x}/k_{x}dt.$$

The movement displacement *c*_x_ required by the compensation lens is

(11) $$c_{x}=a_{x}e=\int_{0}^{t}\omega_{x}e/k_{x}dt.$$

Since the compensation lens is driven by a piezoelectric micro-mechanism, and the output displacement of the driving mechanism is directly proportional to the control signal voltage, let the linear relationship between the output displacement *c_x_* and the control signal voltage Δ*U* be expressed as [36]

(12) $$c_{x}=nd_{33}\Delta Uk_{p}/K_{e}$$

where *n* is the layers of the piezoelectric stack, *d*_33_ is the piezoelectric coefficient, and *k*_p_ is the stiffness of the laminated piezoelectric ceramics.

Since the laminated piezoelectric ceramics can only be connected to positive voltage, in order to realize the positive and negative movement of the micro-mechanism based on the origin, the voltage of the control signal needs to be set to positive bias. Assuming that the motion stroke of the micro-mechanism is ±50 μm, according to the relationship between the output displacement of the micro-mechanism and the voltage, the control voltage signal bias 60 V can be used as the origin of the positioning mechanism. According to the angular velocity at this point, the control voltage of the positioning mechanism is

(13) U=60+ΔU.

Assuming that the relation parameter between the voltage signal output by the gyroscope and angular velocity is *ε*, the voltage Δ*u* corresponding to the rotation angle of the compensation mechanism at *t* time within a period of movement is

(14) $$\Delta u=\varepsilon \int_{0}^{t}\omega_{x}dt.$$

Combining the formulas, the relation between the control voltage increment Δ*U* and the output voltage Δ*u* of the gyroscope at time *t* in a cycle can be obtained

(15) $$\Delta u=\frac{\varepsilon nd_{33}k_{p}k_{x}}{eK_{e}}\Delta U.$$

### 2.5. Design of the Stabilizing Structure

**Figure 11:**
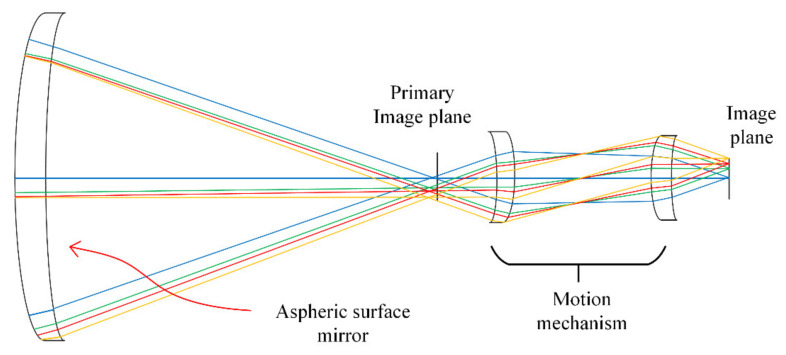
The optical path.

The target object is set at a distance of 1 m from the lens, and the field of view is 5°. The optical path is designed as shown in Figure 11. In order to verify the optical anti-shake by moving lens components, the middle image plane is set in the optical system designed, and a motion mechanism is set between the primary image plane and the secondary image plane. The optical magnification of the motion mechanism is −1. In the image stabilization experiment, when the distance of the optical axis offset by the motion mechanism is d, the position change of the image point before and after the motion is 2*d*. An even aspheric surface is added to balance the phase difference in the former group of lenses for obtaining better imaging quality at the primary image plane.

**Figure 12:**
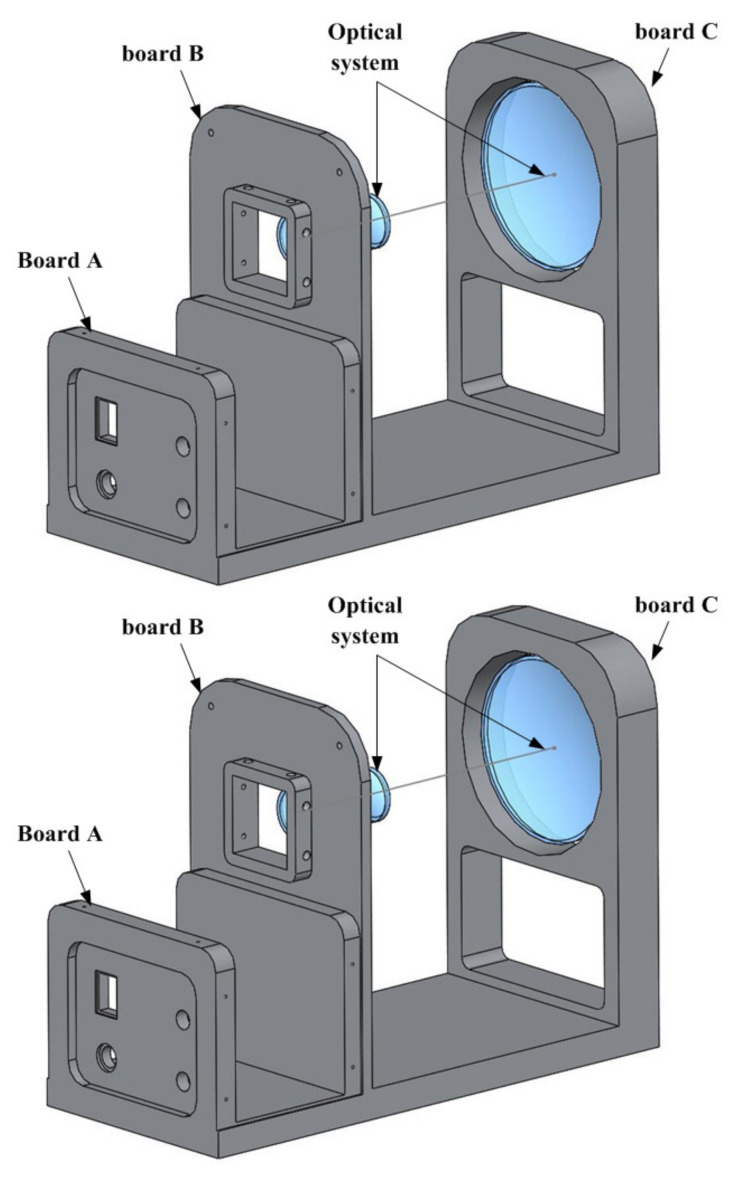
The structure of optical image stabilization system.

According to the working principle of optical image stabilization and the design of the optical system scheme, the structure of the optical image stabilization system is designed as shown in Figure 12. Since the controller generates a lot of heat when it works, the position of the controller can be set at the position of board A behind the infrared camera to avoid the influence on infrared imaging. Considering the imaging position in the optical system, the infrared imager is installed on board B at the position of the secondary image plane in the optical path. 

**Figure 13:**
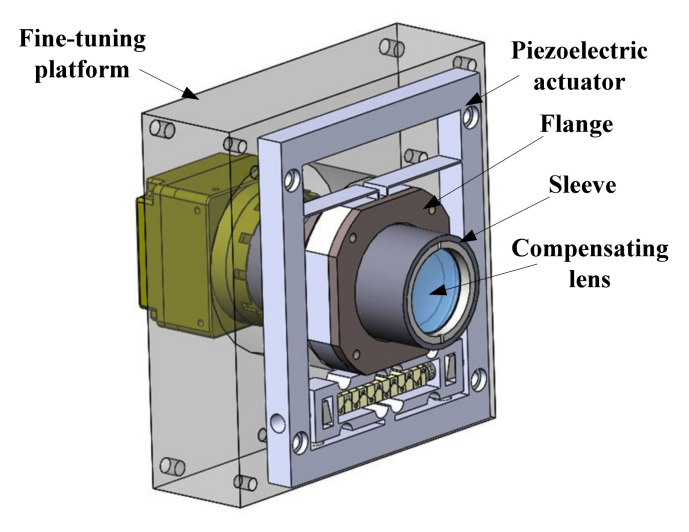
The connection mechanism of the compensating lens.

Since the position of the compensating lens is special in the optical system, the connection way as shown in Figure 13 is designed. When it comes to the installation steps, the compensating lens is installed in the sleeve firstly. Then, the sleeve is installed on the flange through a thread connection, and the flange is fixed on the working platform of the piezoelectric actuator by a screw. The piezoelectric actuator is fixed on the fine-tuning platform next, and finally, the fine-tuning platform is connected to board B of the base.

**Figure 14:**
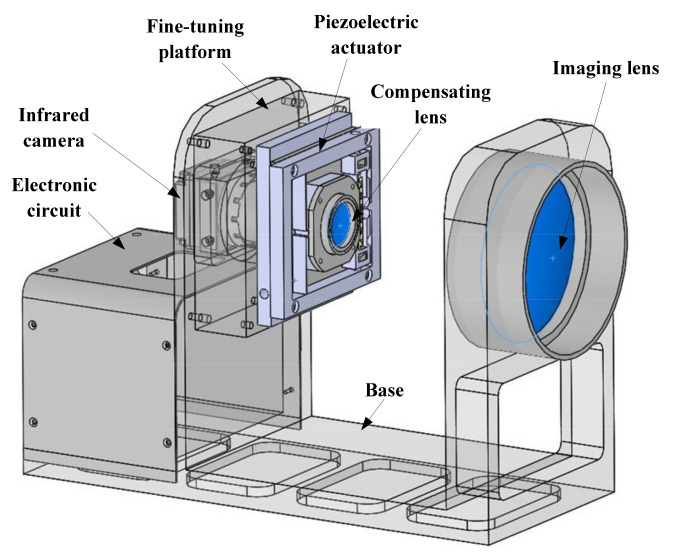
The overall structure of the infrared optical image stabilization system.

As shown in Figure 14, the overall structure of the infrared optical image stabilization system consists of an electronic circuit, infrared camera, two-dimensional fine-tuning platform, piezoelectric actuator, compensating lens, imaging lens, and base. The electronic circuit includes the driving circuit of the actuator and the motion detection device. The infrared camera is an uncooled TC388 infrared imager. The compensating lens is mounted on the piezoelectric actuator. Due to the error in the installation process of the actuator, there is a big deviation between the axis of the compensating lens and the axis of the imaging lens. Thus, a two-dimensional fine-tuning platform is added between the actuator and the base, and the optical axis of the compensating lens can be guaranteed by adjusting the two-dimensional fine-tuning platform to align with the optical axis of the imaging lens.

## 3. Analysis of Experimental Results

**Figure 15:**
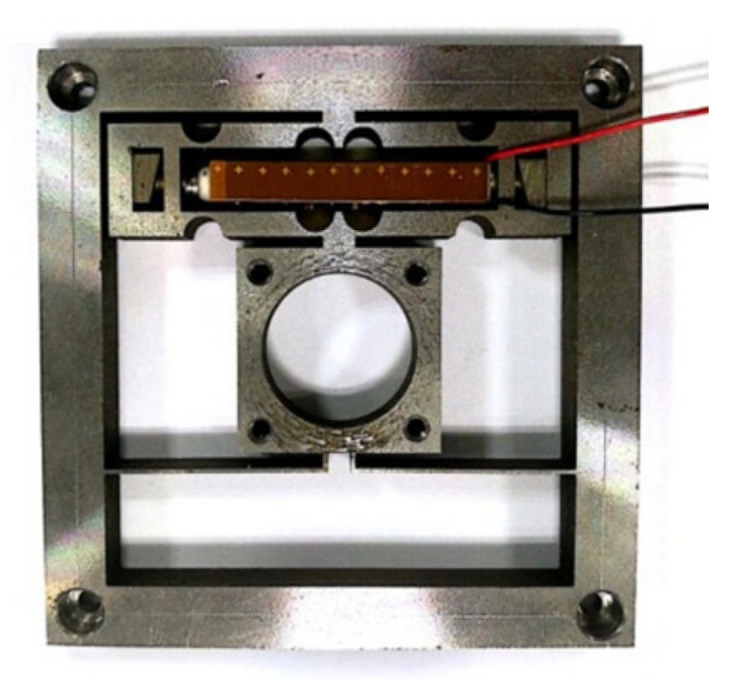
The prototype of the piezoelectric actuator.

The performance of the piezoelectric actuator was tested. The piezoelectric actuator prototype is shown in Figure 15. The piezoelectric ceramics used in the prototype is PK4FYP1 from THORLABS, which has a free stroke of 41 μm at the voltage of 150 V. The dimensions of piezoelectric ceramics are 5.2 mm × 5 mm × 38.1 mm.

**Figure 16:**
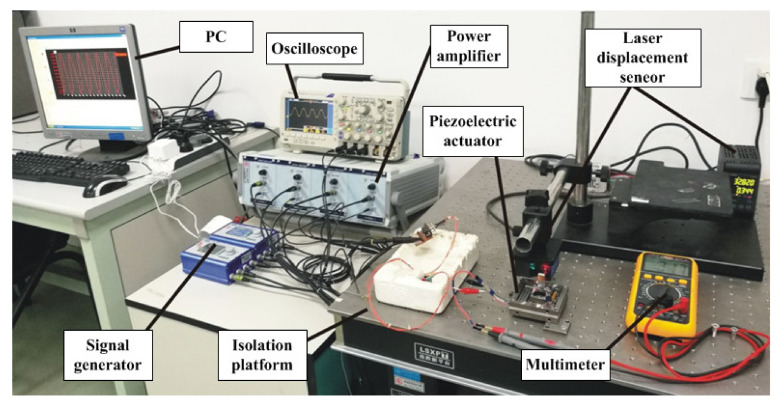
The experiment environment.

The experimental environment is shown in Figure 16. The piezoelectric actuator and the laser displacement sensor are set on the vibration isolation platform. The laser displacement sensor used to measure the displacement was the laser displacement sensor LK-H020 from Keyence Company with the resolution of 20 nm. A voltage signal produced by the signal generator is applied on the actuator through a power amplifier. The multimeter measures the capacitance of piezoelectric ceramics to determine its integrity, and a PC is used to observe output data from the displacement sensor.

### 3.1. Displacement Measurement of Piezoelectric Ceramics

**Figure 17:**
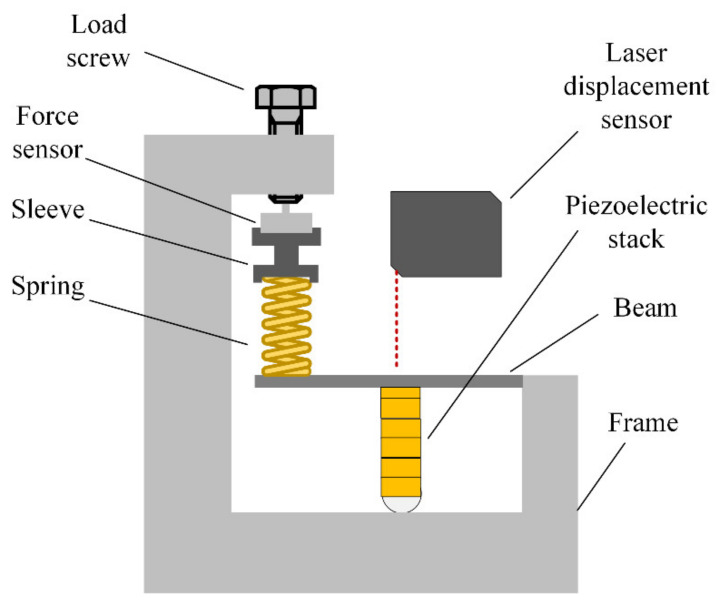
Test system for laminated piezoelectric ceramics.

Firstly, the dynamic performance of the piezoelectric ceramics used was tested. Since a certain preload is required by the piezoelectric ceramics in use, the mechanism as shown in Figure 17 was used for testing. A certain pretightening force can be applied to the laminated piezoelectric ceramics through the pretightening bolt on the pretightening device. Based on the applied preload returned through the pressure sensor, the preload applied in the laminated piezoelectric ceramic is about twice as much as indicated by the load sensor. After applying a sinusoidal voltage signal to the laminated piezoelectric ceramics, the displacement changes of the laminated piezoelectric ceramics can be measured by a laser displacement sensor at the position shown in the picture, and the relevant results can be obtained by collecting the signal.

**Figure 18:**
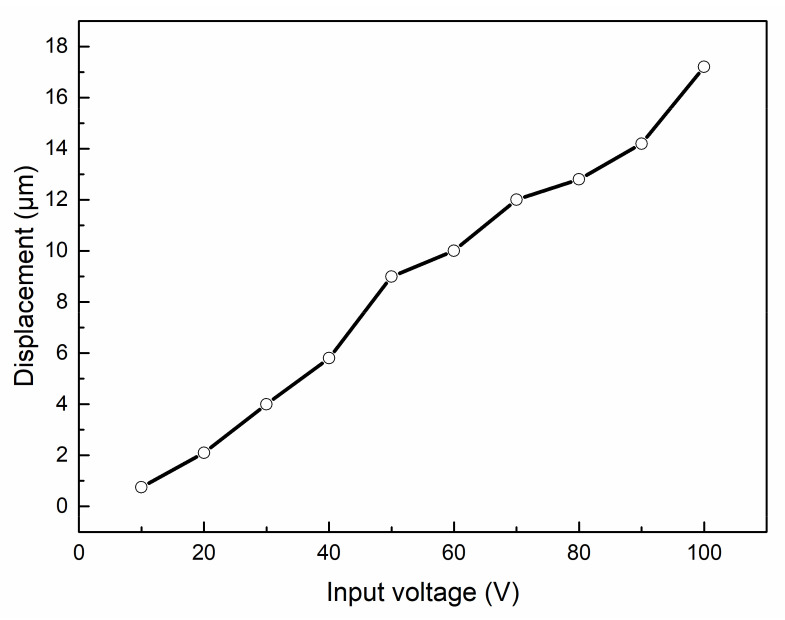
Displacement of the piezoelectric ceramic versus input voltage.

In the actual test, the laminated piezoelectric ceramics were pressed, and electrical signals were input at the positive and negative poles. The voltage-dependent displacement variation of the piezoelectric ceramics was measured by laser displacement sensor, as shown in Figure 18. It can be seen that when the input voltage was 10–100 V, the output displacement of the laminated piezoelectric ceramic changed linearly with the voltage, and the maximum output displacement was about 17 μm.

### 3.2. Displacement Test of the Actuator

**Figure 19:**
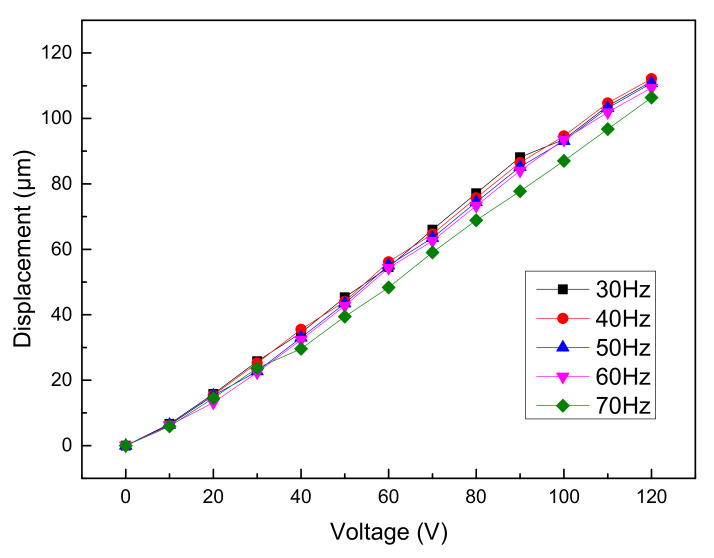
Displacement of the actuator versus input voltage under different frequencies.

The performance of the piezoelectric mechanism was tested by using the test system as shown in the figure, and the sine wave voltage signal was input into the piezoelectric ceramics. To stay away from the resonant mode of the mechanism, the mechanism should be tested and used at low frequencies. By changing the voltage and frequency of the driving signal, the result as shown in Figure 19 can be obtained.

As can be seen from the figure, the output displacement of the micro-motion mechanism changed insignificantly with the increase of input signal frequency, but it increased linearly with the increase of input signal voltage. When the working frequency changes from 30 to 70 Hz, the piezoelectric actuator works stably. Under the same voltage, the output displacement of the actuator changes little with frequency. This result is ideal, because when the frequency changes, only the moving velocity changes, and the step should change with the change of voltage amplitude. The linearity between the output displacement and voltage was good, so the performance curve at the frequency of 50 Hz can be used as the working curve of the image stabilization system. When the input signal voltage was 120 V and the frequency was 50 Hz, the maximum displacement of the piezoelectric micro-motion mechanism was 115 μm. When the input signal was 10 V, the minimum displacement of the mechanism was 8 μm.

According to the above test results, the fitting formula of the output displacement of the piezoelectric micro-actuator and the input signal voltage *U* can be obtained.

(16) $$u_{i}=0.97U$$

The actual amplification ratio *β* of the piezoelectric actuator is 5.7.

(17) $$\beta =\frac{0.97U}{10.1}=5.7$$

The amplification ratio obtained by the test is basically consistent with the simulation result.

### 3.3. Transient Performance Test

**Figure 20:**
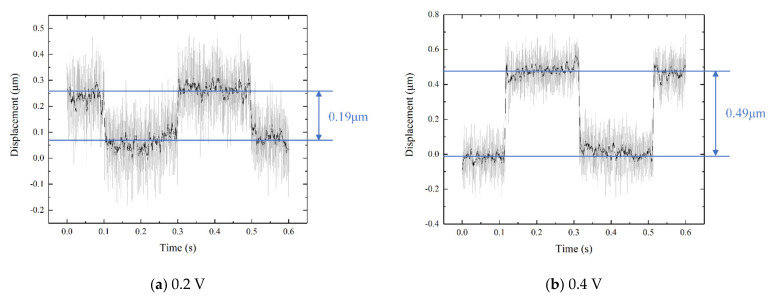

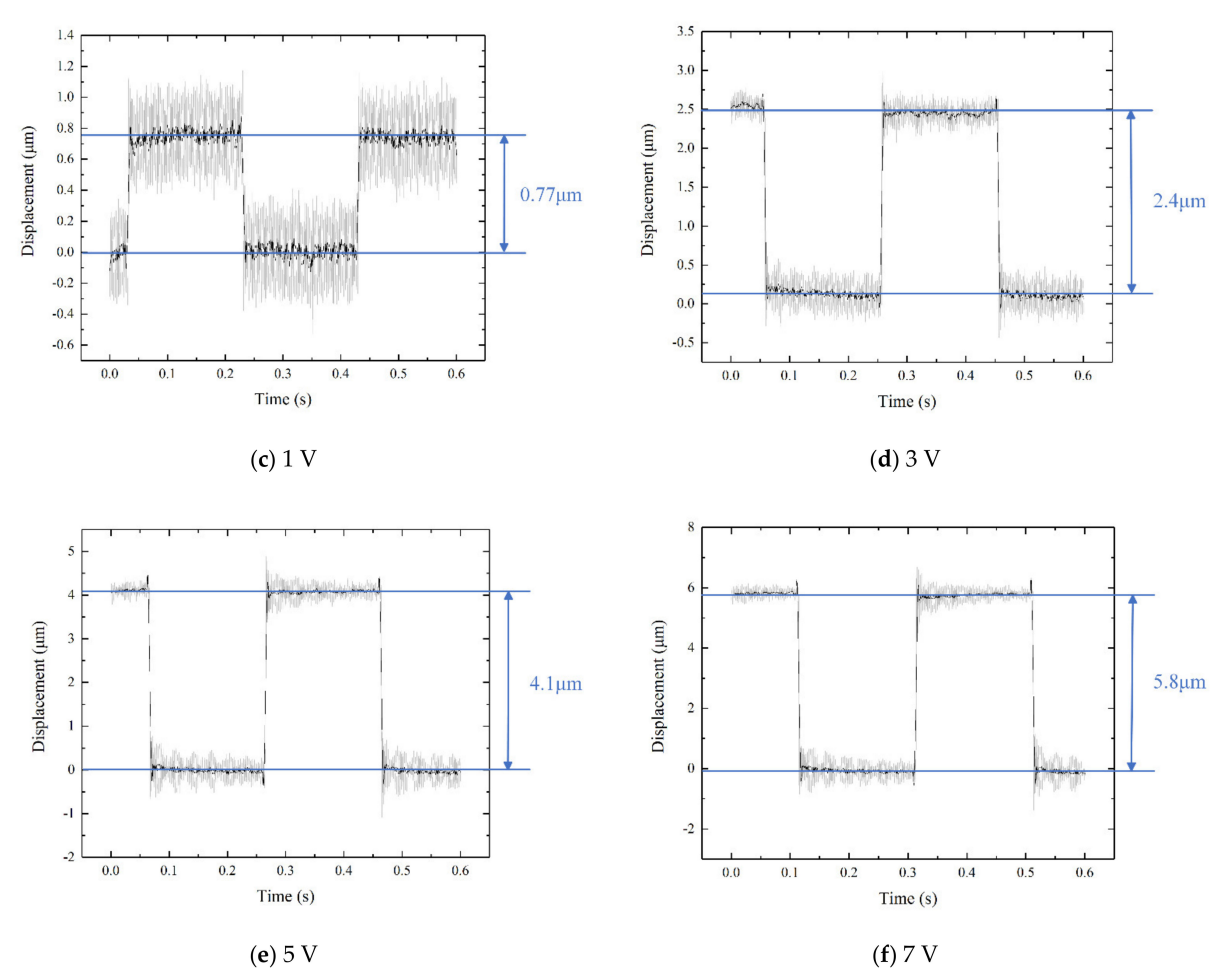
Displacement of the mechanism when the input voltage is (**a**) 0.2 V, (**b**) 0.4 V, (**c**) 1 V, (**d**) 3 V, (**e**) 5 V, and (**f**) 7 V.

Input square wave signals with different voltage amplitudes were used to test the start–stop characteristics and step displacement of the micro-mechanism. The input voltage amplitude was set to 0.2 V, 0.6 V, 1 V, 3 V, 5 V, and 7 V, respectively. The motion displacement of the micro-mechanism was tested using the laser displacement sensor, and the displacement curve was obtained, as shown in Figure 20.

According to the results, when the input voltage was 0.2 V, 0.4 V, and 1 V, the stepping displacement of the mechanism was 0.19 μm, 0.49 μm, and 0.77 μm, respectively. However, at this point, the noise of the single-step micro-motion was large, which basically accounted for 50% or more of the step displacement, which is not suitable for precise positioning. When the input voltage increased to 3 V, 5 V, and 7 V, the stepping displacements of the mechanism were 2.4 μm, 4.1 μm, and 5.8 μm, respectively, and the noise of the single-step micro-motion was relatively small, so the motion adjustment with precision of 10 μm can be achieved without filtering modulation, and the response time can also be approximated to the order of milliseconds. The response time of the actuator depends on the quality of piezoelectric ceramics and the complexity of the structure. The millisecond response time is the average level of piezoelectric structures. In the infrared image stabilization system introduced in this paper, the actuator with a precision of 2.4 μm is enough to realize the real-time displacement adjustment of the compensation lens. In order to increase the output accuracy, a low-pass filter can be added to the circuit to remove burrs.

**Figure 21:**
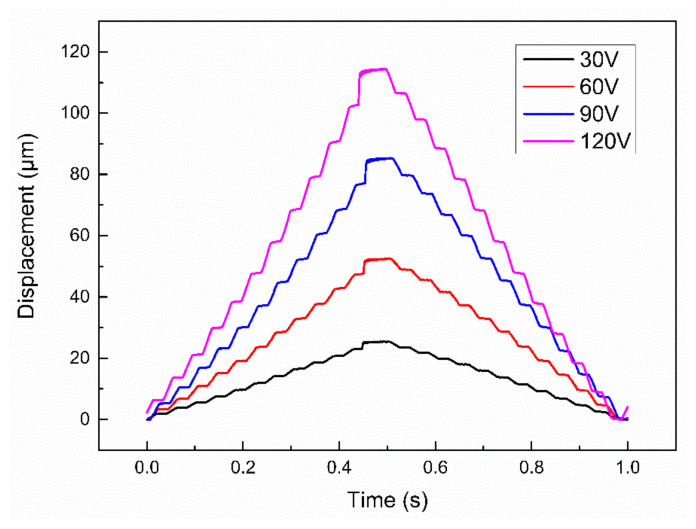
Step displacement of the mechanism when the input voltage is 30 V, 60 V, 90 V, and 120 V.

When the voltage further increased to 30 V, 60 V, 90 V, and 120 V, the displacement curve under the up and down step wave is shown in Figure 21. These results can be utilized as the coarse motion to obtain a large range and high velocity. When the voltage of the step wave increases, the actuator stroke increases from 20 to 115 μm, and the step distance increases from 2.4 to 11 μm. In the process of real-time image stabilization, the piezoelectric mechanism can be controlled to push the compensation lens to a suitable distance by adjusting the input voltage. In the test, it is found that the up and down step distance of the actuator are slightly different, but their errors are within the 10 μm displacement corresponding to one pixel, which has little impact on the image stabilization adjustment. The multiple accumulation error can be eliminated by returning the actuator to the initial position through the initial signal between frames during imaging.

### 3.4. Image Stabilization Test

**Figure 22:**
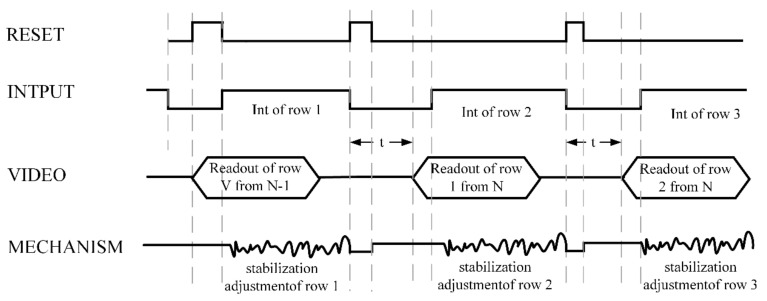
Working sequence diagram of an infrared imaging system.

In order to realize the synchronization function of an image output and imaging system and ensure the real-time image stabilization function in the process of image acquisition, it is necessary to conduct the control design of an imaging sequence. Figure 22 shows the working time sequence diagram of the infrared imager. It can be seen from the figure that the acquisition and output of images are in behavioral units. The acquisition process of each line of images can be regarded as the integration process of energy over time, and output is carried out after each line of images is collected. The working process is as follows: the first line image is collected when the reset signal is received, the first line image is output after a certain interval t, and the instruction is issued to collect the second line image at the same time, and so on to finally complete a frame of image collection. According to the imaging process and image output process of an infrared imager, to output a stable frame of an image under external interference, it needs to drive the image stabilization mechanism during image acquisition to compensate for the imaging offset caused by system jitter. At this point, a synchronization signal is used to transmit the beginning and end of image acquisition. After each frame of the image is collected, the image stabilization mechanism is reset to the origin and waits for the next frame of the image to be collected. The stable output of the image can be realized through such repeated cycles.

**Figure 23:**
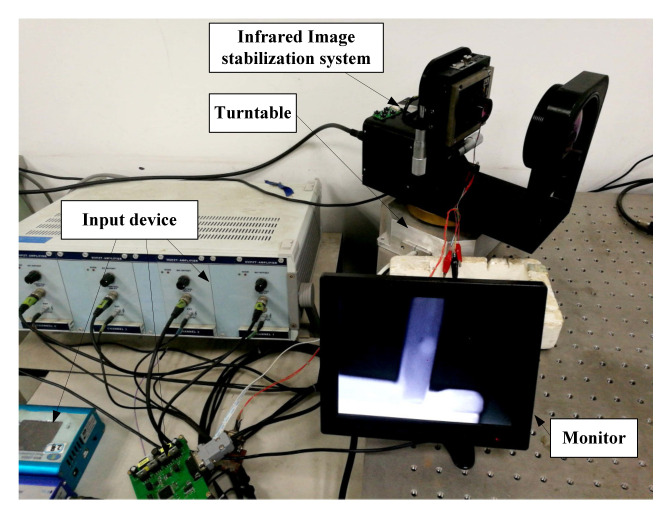
The test system.

The designed infrared image stabilization system is placed on the vibration platform, and the experimental device is shown in Figure 23.

When the image stabilization system is running, the input fluctuation can be detected by the gyroscopic sensor, and the displacement of the compensation lens driven by the piezoelectric actuator can be calculated in real time and input into the piezoelectric ceramics. 

**Figure 24:**
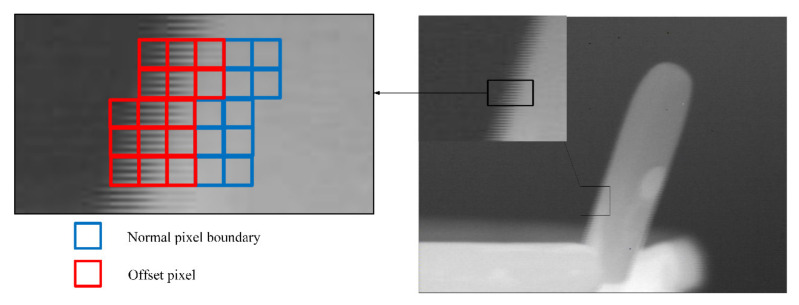
The infrared image under the interference condition.

**Figure 25:**
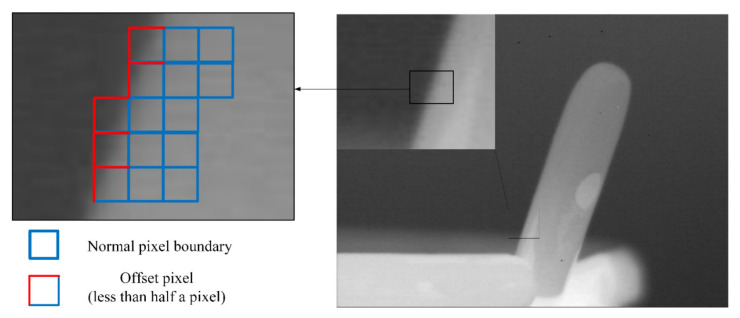
The infrared image after image stabilization.

The infrared imaging image after inputting the fluctuation and opening the image stabilization mechanism in the imaging system is shown in Figure 24 and Figure 25. As shown in Figure 24, when the rotation disturbance is applied to the infrared imaging system through the turntable, the image boundary is obviously blurred. By meshing the boundary, it can be seen that the image offset distance is about two to three pixels under a given rotation disturbance. As shown in Figure 25, when the piezoelectric actuator is used to drive the compensation lens to stabilize the image in the imaging process, the blur of the image boundary is reduced, and the offset distance of the image line is reduced to less than half a pixel.

It can be seen that after the introduction of the image stabilization system, the imaging offset of the imaging system in the fluctuation is reduced by several pixels, thus verifying the feasibility of the image stabilization system of the piezoelectric actuator.

## 4. Conclusions

In this study, an infrared image stabilization system based on a piezoelectric actuator is designed and fabricated. The image stabilization system consists of an electronic circuit, infrared camera, two-dimensional fine-tuning platform, piezoelectric actuator, compensating lens, imaging lens, and base. The compensating lens is mounted on the piezoelectric actuator. A piezoelectric actuator with a bridge-type mechanism is designed to drive the compensating lens to achieve real-time and high-precision micro-displacement action. An equivalent stiffness model is applied to design the actuator. The design parameters from the numerical model are confirmed by the finite element method. A series of actuator experiments are carried out to test the kinetic characteristics. The following conclusions are obtained.

(1) As revealed by the displacement tests, the actuator has a good linearity between the displacement and the different voltage. The maximum output displacement is 115 μm with a voltage of 120 V, and the minimum displacement is 8 μm with a voltage of 10 V. Combined with the test results of laminated piezoelectric ceramics, the amplification ratio of the mechanism is calculated as 5.7.
(2) As shown by the transient performance test on the actuator, when the input voltage increased to 3 V, 5 V, and 7 V, the stepping displacements of the mechanism were 2.4 μm, 4.1 μm, and 5.8 μm, respectively, and the noise of the single-step micro-motion was relatively small, so the motion adjustment with a precision of 10 μm can be achieved without filtering modulation.
(3) The displacement curves under the up and down step wave indicate that when the voltage of the step wave increases, the actuator stroke increases from 20 to 115 μm, and the step distance increases from 2.4 to 11 μm. It is found that the up and down step distance of the actuator has a slight difference, but their errors are within the 10 μm displacement corresponding to one pixel, which has little impact on the image stabilization adjustment.
(4) It can be seen from the image stabilization test, after the introduction of the image stabilization system, the imaging offset of the imaging system in the fluctuation is reduced by several pixels, thus verifying the feasibility of the image stabilization system of the piezoelectric actuator.

It has been successful in obtaining a stable infrared image with the assistance of the image stabilization based on a piezoelectric actuator featuring a simple structure, high resolution, and fast response.

## Figures and Tables

**Table 1:** The main materials and dimensions of the piezoelectric mechanism.

Parameters	Values	
	Metal Elastic Mechanism (Stainless Steel)	Piezoelectric Ceramics
Density (kg/m^3^)	7900	7640
Elasticity modulus (Pa)	2 × 10^11^	-
Poisson’s ratio	0.3	0.31
d_33_ (m/V)	-	7.2 × 10^−10^
Size	92 mm × 78 mm × 8 mm (overall size)	5.2 mm × 5 mm × 38.1 mm
	44 mm × 44 mm × 8 mm (center frame size)	-
	29.5 mm × 0.5 mm × 8 mm (Cantilever beam size)	-
	58 mm × 14 mm × 8 mm (size of piezoelectric ceramic mounting frame)	-
